# Substantial transition to clean household energy mix in rural China

**DOI:** 10.1093/nsr/nwac050

**Published:** 2022-03-14

**Authors:** Guofeng Shen, Rui Xiong, Yanlin Tian, Zhihan Luo, Bahabaike Jiangtulu, Wenjun Meng, Wei Du, Jing Meng, Yuanchen Chen, Bing Xue, Bin Wang, Yonghong Duan, Jia Duo, Fenggui Fan, Lei Huang, Tianzhen Ju, Fenggui Liu, Shunxin Li, Xianli Liu, Yungui Li, Mu Wang, Ying Nan, Bo Pan, Yanfang Pan, Lizhi Wang, Eddy Zeng, Chao Zhan, Yilin Chen, Huizhong Shen, Hefa Cheng, Shu Tao

**Affiliations:** College of Urban and Environmental Sciences, Peking University, Beijing 100871, China; College of Urban and Environmental Sciences, Peking University, Beijing 100871, China; College of Urban and Environmental Sciences, Peking University, Beijing 100871, China; Collaborative Innovation Center of Atmospheric Environment and Equipment Technology, Jiangsu Key Laboratory of Atmospheric Environment Monitoring and Pollution Control (AEMPC), Nanjing University of Information Science and Technology, Nanjing 210044, China; College of Urban and Environmental Sciences, Peking University, Beijing 100871, China; Institute of Reproductive and Child Health, Peking University, Beijing 100191, China; College of Urban and Environmental Sciences, Peking University, Beijing 100871, China; Laboratory of Geographic Information Science, School of Geographic Sciences, East China Normal University, Shanghai 200241, China; The Bartlett School of Sustainable Construction, University College London, London WC1E 7HB, UK; College of Environment, Zhejiang University of Technology, Hangzhou 310014, China; Institute of Applied Ecology, Chinese Academy of Sciences, Shenyang 110016, China; Institute of Reproductive and Child Health, Peking University, Beijing 100191, China; Department of Epidemiology and Biostatistics, School of Public Health, Peking University, Beijing 100191, China; College of Resources and Environment, Shanxi Agricultural University, Jinzhong 030801, China; Xinjiang Key Laboratory of Environmental Pollution and Bioremediation, Xinjiang Institute of Ecology and Geography, Chinese Academy of Sciences, Urumqi 830011, China; National Engineering Technology Research Center for Desert-Oasis Ecological Construction, Xinjiang Institute of Ecology and Geography, Chinese Academy of Sciences, Urumqi 830011, China; University of Chinese Academy of Sciences, Beijing 100049, China; School of Geography and Tourism, Anhui University, Wuhu 241000, China; School of Environment, Nanjing University, Nanjing 210033, China; College of Geography and Environmental Science, Northwest Normal University, Lanzhou 730070, China; College of Geographical Science, Qinghai Normal University, Xining 810008, China; Academy of Plateau Science and Sustainability, Xining 810008, China; College of Chemistry, Chemical Engineering and Environment, Minnan Normal University, Zhangzhou 363000, China; School of Environmental Science and Engineering, Hubei Polytechnic University, Huangshi 435003, China; Department of Environmental Engineering, Southwest University of Science and Technology, Mianyang 621010, China; College of Food Science, Tibet Agricultural and Animal Husbandry University, Linzhi 860000, China; College of Geography and Ocean Sciences, Yanbian University, Yanji 133002, China; Faculty of Environmental Science and Engineering, Kunming University of Science and Technology, Kunming 650500, China; College of Geography and Environmental Science, Henan University, Kaifeng 475001, China; College of Ecology and Environment, Hainan University, Haikou 570228, China; School of Environment, Jinan University, Guangzhou 510632, China; Institute of Coastal Research, Ludong University, Yantai 264025, China; College of Environmental Science and Technology, Southern University of Science and Technology, Shenzhen 518055, China; College of Environmental Science and Technology, Southern University of Science and Technology, Shenzhen 518055, China; College of Urban and Environmental Sciences, Peking University, Beijing 100871, China; College of Urban and Environmental Sciences, Peking University, Beijing 100871, China; College of Environmental Science and Technology, Southern University of Science and Technology, Shenzhen 518055, China

**Keywords:** household energy mix, energy transition, clean heating, modern energy, sustainable development

## Abstract

The household energy mix has significant impacts on human health and climate, as it contributes greatly to many health- and climate-relevant air pollutants. Compared to the well-established urban energy statistical system, the rural household energy statistical system is incomplete and is often associated with high biases. Via a nationwide investigation, this study revealed high contributions to energy supply from coal and biomass fuels in the rural household energy sector, while electricity comprised ∼20%. Stacking (the use of multiple sources of energy) is significant, and the average number of energy types was 2.8 per household. Compared to 2012, the consumption of biomass and coals in 2017 decreased by 45% and 12%, respectively, while the gas consumption amount increased by 204%. Increased gas and decreased coal consumptions were mainly in cooking, while decreased biomass was in both cooking (41%) and heating (59%). The time-sharing fraction of electricity and gases (E&G) for daily cooking grew, reaching 69% in 2017, but for space heating, traditional solid fuels were still dominant, with the national average shared fraction of E&G being only 20%. The non-uniform spatial distribution and the non-linear increase in the fraction of E&G indicated challenges to achieving universal access to modern cooking energy by 2030, particularly in less-developed rural and mountainous areas. In some non-typical heating zones, the increased share of E&G for heating was significant and largely driven by income growth, but in typical heating zones, the time-sharing fraction was <5% and was not significantly increased, except in areas with policy intervention. The intervention policy not only led to dramatic increases in the clean energy fraction for heating but also accelerated the clean cooking transition. Higher income, higher education, younger age, less energy/stove stacking and smaller family size positively impacted the clean energy transition.

## Teaser

In rural China, affordable modern energy was increasingly adopted for cooking, and clean energy carriers were also used more for space heating.

## INTRODUCTION

Clean and sustainable household energy is an important part of Sustainable Development Goal (SDG) 7—affordable and clean energy—and is closely related to other SDGs, such as good health and well-being (SDG 3), climate action (SDG 13), life on land (SDG 15) and gender equality (SDG 5) [1]. Although the global number of people with access to electricity increased to 90% by 2018, 789 million people still did not have electricity [2,3], and nearly 2.8 billion people still heavily relied on traditional solid fuels (coal, crop straws, wood, animal dung, etc.) as their main residential energy sources [2,4].

Household energy is an important foundation of the lives of all people and is closely related to clean air; however, most solid fuel users lack cleaner household energy. In many rural households, traditional solid fuels are still predominant, the burning of which, in rudimentary stoves, releases large amounts of air pollutants into not only ambient but also indoor air, resulting in severe household air pollution [5–8]. Daily average indoor PM_2.5_ concentration in households using solid fuels such as coal, crop straw, wood and animal dung can be as high as several hundred μg per m^3^, 10 times more than the guideline set to protect human health [9–12]. Emissions from the residential sector not only affect air quality and human health, but also regional and global climate change with its significant contribution to many climate forcers like CO_2_, black carbon (BC) and organic aerosols [13–16].

Compared to the well-established statistical systems for energy in sectors such as industry, transportation and agriculture, information on the mix of energy sources used by rural households is very limited, and has high uncertainties and biases because of incomplete statistical data and the high consumption of non-commercial biomass fuels. It was previously found that the International Energy Agency (IEA) and Food and Agriculture Organization (FAO) of the United Nations simply estimated household biomass consumption from crop yields, and significantly overestimated residential biomass consumption in China [17], which consequently hindered accurate estimations of air pollutants and carbon emissions. In addition, the pervasive stacking of multiple energy sources and stoves in homes to meet various daily life demands complicates the accurate quantification of household energy and affects the adoption of modern energy and suspension of the use of dirty solid fuels [18–20].

Our previous study pointed out that from 1992 to 2012, spontaneous transition to modern energy carriers like gas and electricity occurred rapidly for cooking, but very slowly for space heating [17]. Transition to cleaner modern energy in China had significant health benefits associated with reduction of PM_2.5_ exposure [13,21], but also had climate co-benefits due to reduced BC emissions [13]. Energy transition is affected by various factors such as accessibility, affordability and burner type, as well as many non-technical factors like awareness of energy saving and human health protection [22,23]. Cooking energy can transit very quickly, sometimes in just one or two years [24]. Under the fast socio-economical development in China, and especially with the clean heating actions in the northern area to fight severe wintertime pollution and haze epoxides, it is thought that household energy underwent very fast changes that have not been studied yet.

The present study, using a nationwide on-site survey conducted in 2017 on rural household energy source mixes covering all provinces/municipalities in China's mainland (data for Hong Kong, Macao and Taiwan are not available here) with a sample size of ∼57 000 (see Methods), aims to understand the realistic household energy mix for different (cooking and heating) demands, reveal the changing trend in the adoption of relatively clean energy sources like electricity and gases (E&G), and evaluate the roles of natural and socio-economic factors in influencing residential energy transition at the national and individual levels. Obtaining clean household energy for daily cooking and heating is necessary to ensure planetary health and is an important part of achieving sustainable development.

## RESULTS AND DISCUSSION

### Opportunities and challenges in universal access to clean cooking

Energy stacking is very common, as a single energy source can hardly fulfill all needs in a household. On the national average, only 4.2% of households used solely one energy source, while the majority had two or three different energy sources (38.4% and 34.6%, respectively). Energy stacking is closely associated with stove/facility stacking [25], as seen from the significantly positive relationship between the number of energy types and the number of stoves at both the province and municipality levels (Fig. S1). The average number of energy types was 2.8 per household, and the average number of stoves per household was 1.4. Stacked energy use makes the traditional household energy survey approach, which only accounts for the primary fuel type, inappropriate and likely biased in describing household energy profiles. Some efforts have been made to more accurately account for energy use, e.g. by asking households about both the main and supplemental fuels instead of the primary fuel alone [26], or by counting the time used for each energy source; these efforts have been used successfully in a previous study [14,27].

**Figure 1.**
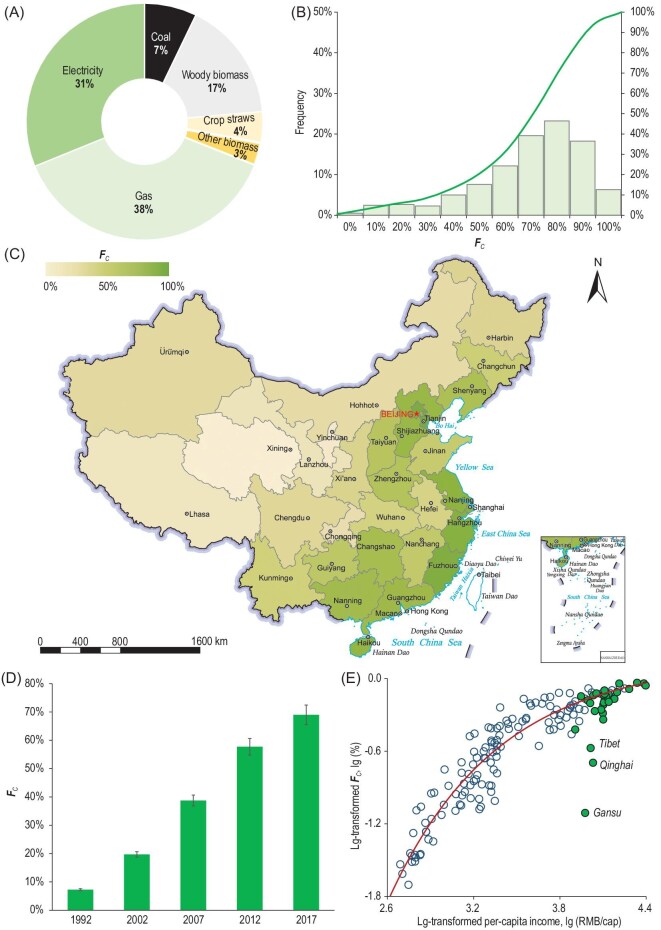
(A) Time-sharing of different energy sources used for cooking in rural China. (B) The frequency distribution of the ***F****_C_*. (C) The spatial distribution of the clean cooking energy source fraction across the country. (D) The national average clean cooking energy source fractions for 1992–2017. (E) The province-level average fractions of clean cooking energy sources against per-capita income for the years 1992–2012 from a previous study [17] and for 2017 from this study (green circles). Note: data from Hong Kong, Macao and Taiwan are temporarily unavailable in this study. Review drawing number: GS(2022)1158.

Figure 1 shows (A) the national average time-sharing fractions of different energy sources used for cooking and (B) the frequency distribution of the time-sharing fraction of cleaner modern energy sources (gas and electricity) for cooking—***F****_C_*. There were only 3.5% rural households with ***F****_C_* ≤ 10%, and nearly one-fourth of rural households used electricity or gaseous fuels predominantly for cooking with***F****_C_* > 90%. The national average ***F****_C_* was 69% in 2017. The transition to modern energy sources for cooking in rural households is very notable, as seen from the***F****_C_* trend from 1992 to 2017 in Fig. 1D; this increasing trend is expected to continue along with economic development. However, the rate of increase was slowing down during this period. In the five years from 2012 to 2017, the national average ***F****_C_* increased from 58% to 69%, i.e., with an increment of 11%, while the increments during the

previous two five-year intervals (2002–2007 and 2007–2012) were 19%.

The dominance of modern energy sources like electricity and gaseous fuels for cooking was evident in most provinces, especially those in the eastern area of China with relatively high socio-economic status (Fig. 1C). In the less-developed western areas, coal and biomass were still dominant fuels for cooking because of their affordability and accessibility. For example, in Shanxi and Inner Mongolia, the rural population burned coal most frequently for cooking, and in Tibet and Sichuan, biomass fuels, including crop straws, animal dung and wood, were available [28] and were the most commonly used cooking fuel. Increased ***F***_C_ values were also clearly revealed at the province level (*p* < 0.05), but the changing rates vary from 1.0% to 4.4% per year across different regions, with relatively higher rates in the developed eastern regions.

Figure 1E plots the province-level ***F****_C_* against the per-capita income and clearly shows a non-linear relationship between the ***F****_C_* and the income levels. The income level can explain ∼88% of the variation in ***F****_C_*. The provincial ***F****_C_* from the present survey generally agreed with the prediction value obtained using a model that was developed based on data from 1992 to 2012 [17]; disagreements were found in the Tibet Autonomous Region and in Qinghai and Gansu provinces. The low ***F****_C_* values obtained for these areas are explained by the limited accessibility of clean cooking energy sources. The spatial diversity and non-linear increases in ***F****_C_* indicate that while universal access to cleaner modern cooking energy by 2030 is hoped for, difficult challenges remain, especially when approaching higher clean cooking energy source fractions. Generally, extensive use of clean energy for cooking in rural households would be achieved earlier in the relatively developed eastern area than in the middle and western areas—a cause of concern with regard to inequality in access to clean cooking energy sources and, consequently, clean air.

### Spatially distinct paths to clean heating

**Figure 2.**
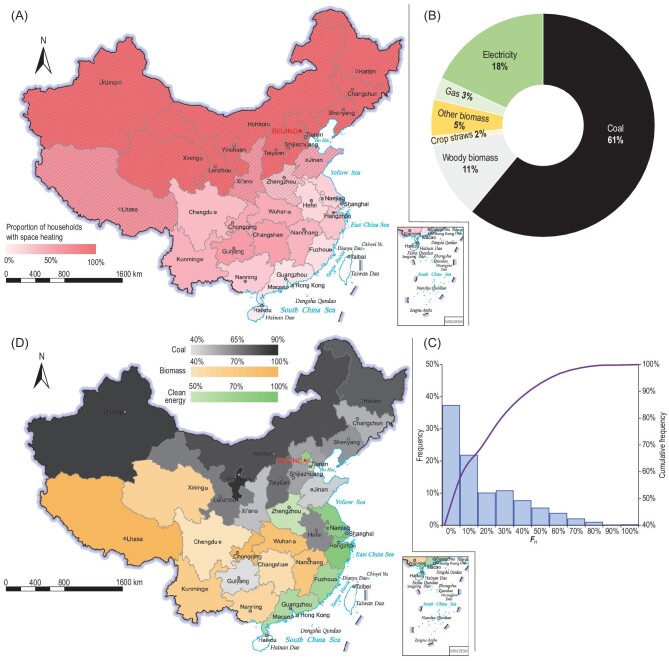
(A) Proportion of households with space heating. (B) The time-sharing of cleaner household energy sources (gas and electricity) for heating. (C) Frequency distribution of the***F****_H_* values. (D) The most widely used energy source (coal, biomass or clean energy) for space heating and its time-sharing fraction. Note: data from Hong Kong, Macao and Taiwan are temporarily unavailable in this study. Review drawing number: GS(2022)1158.

Heating in cold seasons is becoming more common. This occurs not only in typical heating zones, i.e. in the northern and southwestern plateau areas, but also in many eastern and southeastern areas (Fig. 2A), with different heating durations and methods [29]. Of the rural households surveyed, 61% had indoor space heating during cold seasons, which was much higher than the proportion before the 2000s [17]. In the non-typical heating zone, e.g. provinces south of the Huai River (Huai River Policy is a national policy that was instituted during the 1950s and provided free or heavily subsidized indoor space heating during the winter to cities north of the Huai River but not to those to the south**)** [30], more households started to heat houses in cold seasons, mostly by using gas or electricity. Of the 61% of rural households with heating in cold seasons, 70% primarily burned coal or woody fuels in home stoves, while the other 27% used electricity more frequently than other energy sources (Fig. S2). In these households, coal heating stoves were kept and not fully suspended [18]. Thus, if multiple energy uses were taken into consideration, the share of coal was higher (61% vs. 41%) and the share of electricity was lower (18% vs. 27%) than the proportions estimated from the survey on a single primary energy approach.

For the mix of energy sources used for heating, there is high reliance on traditional solid fuels in most rural households. The time-sharing fractions of coal, wood, crop straws and other biomass fuels in heating *Kang* (bed) were 40%, 24%, 18% and 18%, respectively. Electric *Kang* was mentioned in some media reports, and concerns regarding its safety were often discussed [31]. We did not see this specific source in the field survey because the electric *Kang* was still in its pilot stage in very few homes and was not yet produced on a large scale. In other heating facilities not using *Kang,* the time-sharing fractions of coal, biomass, gas and electricity were 41%, 18%, 3% and 18%, respectively (Fig. 2B). The frequency distribution of the time-sharing fraction of cleaner modern energy (E&G) for heating (***F****_H_*) shows that the majority had ***F****_H_* < 50%, and <2% of rural households had ***F****_H_* ≥ 80% (Fig. 2C). In relatively developed eastern areas such as Shanghai, Jiangsu, Zhejiang and Fujian, where there was no central heating, electricity or gas energy sources were most frequently used for space heating (Fig. 2D). In southwestern areas (i.e. Tibet, Chongqing and Sichuan) and most south-central area (i.e. Hubei, Hunan and Guangxi), people had high usages of biomass for heating because of relatively rich biomass resources in these areas [32,33]. In the northern region, coal was the most widely used heating energy source, with a time-sharing fraction higher than 70% or even >90% in the cold northeastern area. Notably, in the BTH area (including Beijing, Tianjin and Hebei), the usage of coals for heating was lower than that in other northern provinces. This is attributed to the clean heating campaign launched in 2016/2017 in this area, which was also known as coal-to-gas/electricity or the coal ban policy [34,35]. Studies have shown that the policy resulted in large reductions in air pollutant emissions and significant health benefits [35–37].

**Figure 3.**
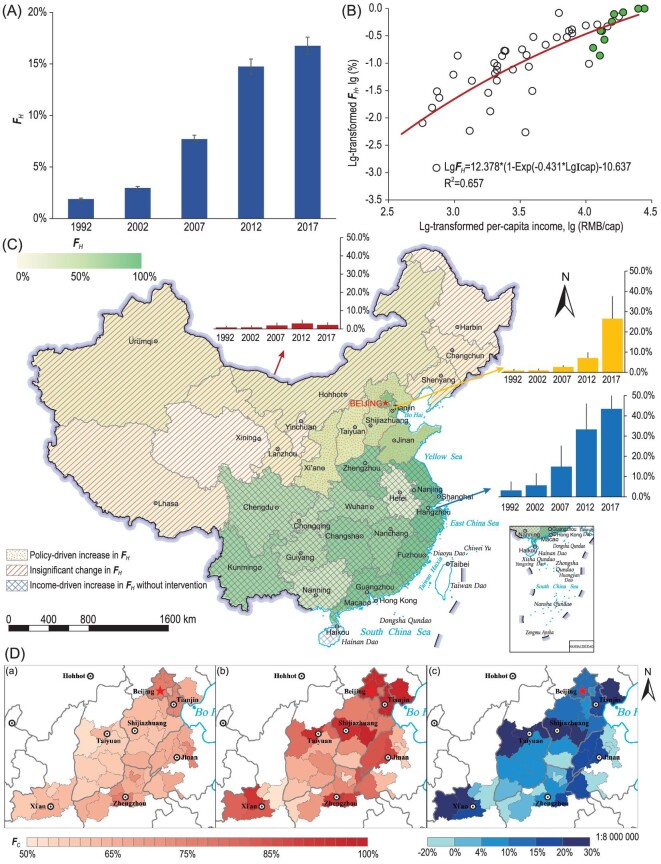
(A) The national average time-sharing fraction of clean heating energy sources in rural areas from 1992 to 2017. (B) The relationship between the ***F****_H_* and per-capita income (**I**cap) for provinces in non-typical heating zones in eastern and south-central regions. (C) Spatial distribution of the ***F****_H_* in 2017. (D) Difference (c) between the predicted ***F****_C_* (a) and the real obtained values (b) in 2017 for areas with a clean energy intervention policy. Note: data from Hong Kong, Macao and Taiwan are temporarily unavailable in this study. Review drawing number: GS(2022)1158.

In the past nearly 20 years, the rural household heating energy mix has also been getting cleaner (Fig. 3A). On the national scale, the national average ***F****_H_* was only 2% in 2002 [17], and reached 17% in 2017; however, the increasing rate of ***F****_H_* without intervention was much lower than that of cooking energy. There are substantial differences in heating demands, natural resources, socio-economical development levels and subsidy policies among different areas, leading to different paths for switching to cleaner modern heating energy sources. Three different transition paths were identified: **A) *Income-driven increase without intervention***—the ***F****_H_* gradually increased along with the increasing household income level. This occurred mainly in provinces from the non-typical heating zones located in southern and eastern areas. In these areas, income can explain 65% of the variation in ***F****_H_* (Fig. 3B). The clean transition rates were 3.7%∼6.2% per year in relatively developed eastern provinces where the provincial ***F****_H_* was close to 60% and >80% in some areas (Fig. 3C). The transition speeds were 1.4%∼2.4% per year in the provinces located in south and south-central areas, such as Sichuan, Yunnan and Hunan, where the provincial ***F****_H_* values were ∼30%–40% in 2017. **B) *Insignificant uptake of cleaner modern energy***—the ***F****_H_* was very low (<5%) and was not significantly different from the values reported in previous years. Solid fuels, especially coals, were still extensively used. This type was mainly found in the northeastern area (Heilongjiang, Jilin and Liaoning) and western provinces, including the plateau area (Qinghai and Tibet) (Fig. 3C). **C) *Policy-driven clean heating transition***—remarkable increases in the ***F****_H_* between 2012 and 2017 but not in years prior to 2012 were found in the BTH region and the Fenwei Plain (covering Shanxi and Shaanxi provinces). The ***F****_H_* values in these provinces in 2012 were close to those in nearby provinces; for example, values were only 5%–7% in BTH but increased dramatically to 80%, 52% and 26% in 2017, respectively. This was due to the national intervention plans for clean heating in the northern region [35,38]. The Clean Heating campaign planned to replace coal sources with either electricity or pipelined natural gas for heating, and the targeted penetration rates were 60% in rural areas and 100% in urban areas by 2021 in the 28 municipalities located in the BTH area [39]. It was reported that by 2019, in almost all cities, the goal of 100% in urban households was achieved, and in rural areas, the average rate was close to 60% but varied from 38% to 97% [40]. Before this national campaign was officially launched in 2017 with definite targets, pathways and subsidy polices, some municipalities started this work earlier. For example, in Beijing, preliminary survey data showed that in 2016 and 2017, there were nearly 760 villages and 1693 villages, respectively, changing from coal to gas or electricity for home heating. A similar program was also implemented in the Fenwei Plain, mainly the 12 cities in Shanxi and Shaanxi provinces.

Among provinces with policy-driven clean heating transition (Group A) or income-driven increase without intervention (Group C), the province-level ***F****_C_* positively correlated with the ***F****_H_* (Fig. S3), while in other provinces (Group B), there was no such relationship since the ***F****_H_* was extremely low. The clean heating intervention in the northern area not only resulted in the rapid uptake of clean energy sources for heating but also significantly accelerated the transition to clean energy sources for cooking. As shown in Fig. 3D, the measured ***F****_C_* was significantly higher than the predicted ***F****_C_* with no intervention [17]. The city-level ***F****_C_* in cities with the intervention policy had an increment of, on the regional average, 11% (7%–15% as 95% CI), whereas in other cities from the same region but with no intervention, the measured ***F****_C_* was close to the previous prediction (*p* > 0.10).

### Dominance of, but significant decline in, solid fuel consumption amounts

The total rural residential consumption of electricity obtained from the survey was 1.17 × 10^6^ TJ in 2017. Fuels, especially solid fuels including coal and biomass, contributed nearly 80% of the total rural residential energy. The annual electricity consumption was 1.83 GJ/capita, varying from 0.49 to 5.35 GJ/capita in different provinces. This value was ∼20% lower than the value of 2.32 GJ /capita reported in the national statistics covering both rural and urban residents [41]. Many studies have discussed the fact that urban residential electricity consumption was higher than that in rural households due to there being more home appliances in urban areas and electricity-saving practices in rural families that lower costs [42,43]. Per-household electricity consumption was negatively correlated with the consumption of fuels (*p* < 0.001) and positively correlated with income (*r* = 0.577, *p =* 0.001). Significant impacts of income with different magnitudes across regions have been reported in past studies [44–46]. For instance, from a metered residential data set in Shanghai [46], it is estimated that annual residential electricity consumption was 7.20–10.9 GJ per household, which was higher in high-income groups. It was reported that in Jiangsu, rural residential electricity consumption increased by 0.063%, while rural disposable income per capita increased by 1% [47].

The total rural residential consumption of coal, biomass and gas fuels, in mass units, was 84.40 (80.81–88.00 as the interquartile range), 180.07 (175.13–185.55) and 13.74 (13.62–17.83) Tg, respectively. On the national average, ∼80% of biomass was used for cooking (Fig. S4). For coal, the consumption amounts for cooking and heating were 38% and 60% of the total, respectively, indicating that a high proportion was used for heating; this result was more obvious in the north, where the percentage of coals used for heating was 65%–87% of the total residential coal consumption in some areas. Gas was still predominately used for cooking, with the gas used for heating accounting for only 1% of the total gas consumption. Relatively higher utilizations of gas for heating were found in the BTH area, in which the consumption amounts of gas for heating comprised up to 6%–9% of the total residential gas in these areas. The BTH region was the first targeted area when the coal ban policy was implemented, leading to a significant proportion of rural households changing from coal heating to gas or electricity heating.

The total annual residential consumption of all fuels, including coal, biomass and gas fuels, in thermal units, was 6.46 × 10^6^ (6.18 × 10^6^ − 6.75 × 10^6^) TJ, with 44%, 46% and 11% of this total sourced from coals, biomass fuels and gases, respectively. Provincial fuel consumption varied from 1.81 × 10^4^ to 4.65 × 10^5^ TJ (Fig. S5). Provincial fuel consumption was positively correlated with the total number of households (*r* = 0.603, *p* < 0.001) (Fig. S6A), which explained only ∼40% of the variation in total fuel consumption. Per-household fuel consumption varied largely among regions. Per-household fuel consumption correlated with the fraction of heating energy to total residential fuel energy (*r* = 0.767, *p* < 0.001) and positively correlated with the solid fuel fraction (*r* = 0.909, *p* < 0.001) (Fig. S6B), indicating that heating activity, especially the use of solid fuels, significantly increased household energy consumption amounts. This is a main reason for the high fuel energy consumption in most northern provinces. In the less-developed western areas, such as Tibet, Qinghai and Ningxia, per-household fuel consumption was higher than that in other areas because of longer heating durations in high-altitude cold areas, larger family sizes, high reliance on traditional solid fuels and relatively lower energy utilization efficiency; however, owing to the small populations in these regions, the provincial total fuel consumption was lower than that in the other areas (Fig. S5).

**Figure 4.**
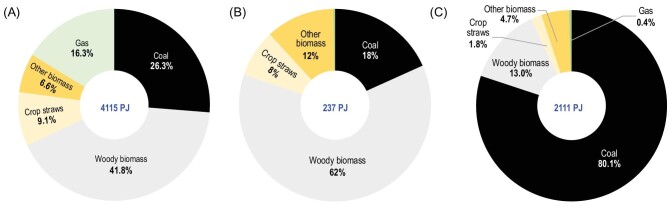
Contributions of different energy types in energy units (petajoules), including coal, crop straw, woody material, other biomass fuels and gases, consumed for daily (A) cooking activities, (B) preparation of animal foods and (C) space heating in China. Note that the circle size is not proportional to the consumption amounts shown in the centers of the circles.

Regarding the energy consumed in different activities, 64% of energy was consumed for cooking and 30% was consumed to meet the heating demand, but these percentages varied across the country. In the northern, northeastern and northwestern regions, 40%–65% of the total energy was used for heating, while in the eastern coastal and southern areas, over 80% of the residential energy coming from fuels was used for cooking. For cooking, coal, biomass and gas fuels contributed 26%, 58% and 16% of the total energy used (Fig. 4A). Although the time-sharing fraction of gas was much higher and its calorific values were higher than those of solid fuels, coal and biomass fuels in home stoves usually have lower energy efficiencies, resulting in very high consumption amounts. In relatively developed municipalities and provinces, the contribution of gas to the total cooking energy could be as high as 40%–50%. For heating, on the national average, ∼80% of the heating energy was supplied by coal, followed by biomass. However, this also varied greatly across regions. In the northern area, coals contributed over 80% and sometimes as high as 90% of the total heating energy. In the central-south and southwestern areas, such as Fujian, Hubei, Chongqing, Sichuan, Yunnan and Tibet, biomass made up the majority, up to 90% or more, of the total household heating energy. Energy sourced from gas fuels in heating activities, on the national scale, was nearly negligible, with a contribution of only 0.4%. In the BTH area, gas contributions were significantly higher, at 1.6%–4.6% of the total residential heating energy.

**Figure 5.**
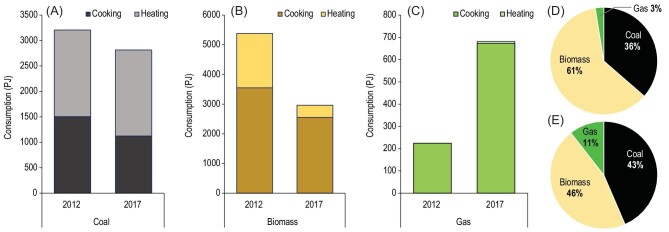
Comparison of rural household (A) coal, (B) biomass and (C) gas consumption in energy units (petajoules) between 2012 and 2017 and the shares of three different energy types in the total rural residential consumption in (D) 2012 and (E) 2017.

Compared to the rural residential fuel consumption of 8810 PJ in 2012, the rural residential fuel consumption in 2017 (6460 PJ) was lower by ∼27%. The rural residential fuel consumption totals were 14 800 and 11 800 PJ in 2002 and 2007, respectively [17], showing a clear decreasing trend (*p* < 0.01) in the total rural residential fuel consumption, at a rate of −560 ± 23 (−460–−659 as 95% CI) PJ/year. Between 2012 and 2017, biomass fuel consumption was reduced by 2410 PJ (990 PJ in cooking activity and 1420 PJ for heating use) and coal consumption was reduced by 392 PJ (381 PJ for cooking and 11 PJ for heating use), whereas gas consumption increased by 458 PJ (449.5 PJ for cooking and 8.6 PJ for heating), resulting in a net reduction of 2350 PJ. Consequently, because of different changes among fuel types, the share of gas in the total rural residential fuel consumption increased from ∼3% in 2012 to 11% in 2017, while the shares of biomass and coal changed from 61% and 36% in 2012 to 46% and 44% in 2017, respectively (Fig. 5). This is consistent with the increased time fractions of clean energy sources and rapid transition to clean energy for cooking activities compared with that for heating activity. The same trend of increased gas fuel with decreased coal and biomass consumption was evidenced at the provincial level but was observed at different magnitudes among provinces.

### Influencing factors at the national and household levels

China's household energy transition is affected by several subjective and external influencing factors [18,48–50]. Income (positive) and heating degree days (HDDs, negative) were found to be the two most significant factors affecting ***F****_C_* and ***F****_H_* [17], and for ***F****_H_*, coal production was another important but negative influencing factor. A survey from three provinces in China showed that younger age, higher income and a smaller household were significantly associated with clean fuel uptake [18]. Here, several associated factors possibly affecting affordability and accessibility, including those from the survey directly (number of cooking and heating energy sources, family size, income, etc.) and collected from national statistics (provincial coal production and consumption, forest coverage, biomass production, etc.), were studied for their impacts on the clean cooking or heating energy fractions. The family income data obtained in the survey were positively correlated with rural disposable income from the national statistical yearbook (*r* = 0.828, *p* < 0.001), but the former was significantly lower (*p* < 0.001) by ∼10%–60%. In many questionnaire studies, income data might be less accurate due to privacy reasons. However, the good agreement on spatial variations indicated that these data could be used to analyze the association between the clean energy fraction and incomes in general but not in quantitative prediction models.

The province-level ***F****_C_* was positively correlated with family income per household (*r* = 0.610, *p* < 0.001) and negatively correlated with the amount of cooking energy (*r* = −0.596, *p* < 0.001) and HDDs (*r* = −0.524, *p* = 0.002). Its correlation coefficient with family size was negative but not statistically significant (*r* = −0.149, *p* > 0.05). As mentioned above (Fig. 1E), the association of ***F****_C_* with income was non-linear, with the increase slowing down when ***F****_C_* increased. ***F****_H_* was negatively correlated with the amount of heating energy (*r* = −0.355, *p* = 0.050) and HDDs (*r* = −0.319, *p* = 0.080) and generally positively correlated with income (*r* = 0.563, *p* = 0.001), but this correlation was more significant in provinces in non-typical heating zones (Fig. 3B). In provinces in the typical heating zone, except for the areas with clean heating intervention action, coal and biomass were still the dominant heating fuels, and the income explained only 30% of the variation in ***F****_H_*, with HDDs explaining another 10%. Again, the influence of family size on ***F****_C_* was negative but not statistically significant (*r* = −0.140, *p* > 0.05). For the other variables analyzed (coal production, forest area, crop area, grain yield), the correlations were not statistically significant (*p* > 0.05).

At the household level, the double-hurdle model results confirmed that family income was a significant positive factor in increasing the usage fraction of clean energy, and HDDs negatively affected ***F****_C_* and ***F****_H_* (Table 1). In large families, the ***F****_C_* and ***F****_H_* values tended to be low. This was explained by the fact that in households with more people, multiple different energy sources were used to meet different activity demands. The numbers of energy types and stoves were both negatively correlated with ***F****_C_* and ***F****_H_*. Age was negatively correlated with ***F****_C_* and ***F****_H_*_,_, suggesting that younger people preferred to use, or had better access to, modern energy sources. The percentage of male residents was not significantly associated with ***F****_C_* or ***F****_H_*. An impact of education level was found only for clean cooking energy but not for heating energy. In the more highly educated groups, there was higher usage of clean cooking energy. The size of a household was not a significant influencing factor in ***F****_C_* but appeared to be positively associated with ***F****_H_*. This is because rural families with higher ***F****_H_* were mostly located in the relatively richer eastern regions of the country, and the floor areas in those homes were large in general. These phenomena were generally consistent with previous study findings. For example, a previous study from Guangxi, Shanxi and Beijing showed that having a higher income, being younger and being more highly educated were enablers in the adoption of clean energy sources, and switching to modern energy tended to occur with life transitions like retirement or the death of a spouse, which was thought to be associated with the adoption of clean energy sources in smaller households [18]. From the Chinese General Social Survey of 2015 across 12 provinces [51], and by using the Tobit regression model, a significant negative relationship between education level and share of biomass was reported, and a high level of education was found to have a positive effect on the shares of liquefied petroleum gas (LPG) and electricity [52].

**Table 1.** Impacts of associated factors in determining the fraction of clean household energy sources (gas and electricity) for cooking (***F****_C_*) and heating (***F****_H_*) in China. Results are from the double-hurdle model.

	***F***_*C*_		***F***_*H*_	
	Estimate	*P-*value^h^	Estimate	*P-*value

Region (ref. North region)^a^				
Northeast region	−0.080	<0.001^***^	−0.037	0.448
East region	−0.123	<0.001^***^	0.006	0.635
South-central region	−0.148	<0.001^***^	−0.100	<0.001^***^
Southwest region	−0.398	<0.001^***^	−0.091	<0.001^***^
Northwest region	−0.439	<0.001^***^	−0.018	0.033^*^
Family size	−0.020	<0.001^***^	−0.028	<0.001^***^
Number of energy types	−0.174	<0.001^***^	−0.045	<0.001^***^
Number of stoves	−0.013	0.003^**^	−0.011	0.087
HDDs	−7.5 × 10^–5^	<0.001^***^	−1.6 × 10^–3^	<0.001^***^
Income^b^	7.5 × 10^–7^	<0.001^***^	8.0 × 10^–7^	<0.001^***^
Age^c^	−0.003	<0.001^***^	−0.001	0.024^*^
Months at home^d^	0.002	<0.001^***^	0.001	<0.001^***^
Male members^e^	−0.017	0.269	0.089	<0.001^***^
Highest education level (ref. no school)				
Primary school	0.024	0.003^**^	−0.010	0.312
Middle school	0.049	<0.001^***^	−0.016	0.224
High school	0.125	<0.001^***^	−0.059	0.012^*^
College and above	0.112	0.035^*^	−0.009	0.855
Female cook	0.006	0.494	0.048	<0.001^***^
Education level of main cook (ref. no school)				
Primary school	0.063	<0.001^***^	−0.003	0.804
Middle school	0.082	<0.001^***^	0.018	0.200
High school	0.086	<0.001^***^	0.005	0.765
College and above	0.069	0.016^*^	0.035	0.238
Farming occupation^f^	−0.071	<0.001^***^	−0.020	0.015^*^
House space area	−1.7 × 10^–5^	0.596	1.8 × 10^–4^	<0.001^***^
House age	−1.5 × 10^–4^	0.016^*^	2.6 × 10^–4^	0.084
Modern household appliances^g^	0.011	<0.001^***^	0.001	0.097

^a^The country is grouped into six regions including North, Northeast, East, South central, Southwest and Northwest (https://en.wikipedia.org/wiki/List_of_regions_of_China). ^ b^The household annual income from the questionnaire. ^ c^The average age of all family members. ^ d^Total months at home of all family members. ^ e^The percentage of male family members. ^ f^The occupation of the main cook—farming or non-farming. ^ g^The total number of household appliances and vehicles including car, motorcycle, electric vehicle, washing machine, refrigerator, TV, air conditioner and computer. ^h^ Significant at the 0.05 (^*^), 0.01 (^**^) and 0.001 (^***^) levels.

These factors affected not only the usage frequency of clean energy sources but also had varying effects on household fuel and electricity consumption amounts (Table S1). Larger families with multiple energy types and stoves consumed more fuels. Electricity consumption was higher in homes with more people and fewer stoves. Highly educated groups consumed less fuel but more electricity than those with lower education levels. Younger groups consumed more electricity, while the older groups consumed more fuels. Household income was negatively associated with fuel consumption and positively associated with electricity consumption. Analyses using statistical yearbook data have shown that income is a critical factor determining rural residential commercial energy consumption [53].

### Summary and implications

In comparison with other energy sectors, information on residential energy patterns and consumption is often associated with relatively higher uncertainties and biases, as statistical data are incomplete and there is a high consumption of non-commercial biomass fuels. From a national home survey and questionnaires, this study found a substantial adoption of gas fuels and electricity for daily cooking activities; these are relatively clean compared to coal and biomass fuels. A significant and continuously changing trend over the past two decades was revealed on the national and provincial scales. The clean cooking energy transition generally occurred without official intervention. In contrast, although the time fraction of electricity or gas for heating increased on the national scale, this increase was mainly due to the adoption of clean energy sources in southern non-typical heating areas, and due to clean heating intervention campaigns in only several specific provinces, while in the other typical heating areas from northern and western areas with high heating demands, coal and biomass were still the major heating fuels and the fraction of gas or electricity in heating was very small. Therefore, without effective interventions, clean heating in rural areas would be difficult to achieve, especially in poor northern and western areas. Income was a significant factor influencing cleaner modern household energy adoption, and younger age, higher education level and smaller family size were associated with longer usage of clean energy. Of course, the lack of clean modern energy is not just a problem of economic conditions. Resources, accessibility, behavioral habits and culture should also be considered in the promotion of cleaner cooking and heating approaches.

In terms of mass and energy units, solid biomass and coal were the major fuels used for daily cooking and heating. The residential consumption of gas increased, and most of this consumption was used for cooking, but overall, gas only contributed to approximately one-tenth of the total residential energy. Biomass and coal consumption decreased, especially biomass; however, biomass and coal still provided most of the total residential cooking and heating energy. High consumption of these solid fuels produces large amounts of air pollutants causing a health threat for the population. The overall air pollution exposure is determined not only by ambient air pollution but also by indoor exposure; therefore, when both indoor and outdoor exposure are considered, residential solid fuels are undoubtedly the largest human health risk source. The impact on human health due to inhalation exposure associated with residential solid fuel use were found to be amplified from <10% contribution in energy supply to nearly 70% of the PM2.5-associated premature deaths [7]. The suspension of solid fuel use has been reported to have significant health and climate benefits [13,18,54,55]. Although more people have access to clean and modern energy sources, due to widespread energy and stove stacking, the extensive use of modern energy in the residential sector is limited, especially when heating activities are also considered together with cooking. It is necessary to note that even for gas and electricity, from coal-fired power plants, there are emissions, and considerable amounts of air pollutants are produced either during the energy production phase or from the burning process [56,57]. However, these emissions are much smaller than those that result from the burning of traditional solid fuels in inefficient residential stoves. The substantial adoption of clean, efficient, affordable household energy will greatly benefit air quality and human health.

A systematic evaluation of the real-world household energy mix is important in order to better understand residential energy transition characteristics and to support the decision-making process. Partially learning from the previous survey in 2012 [17], the present study had a larger sample size, and several limitations in the previous survey were addressed. The stratified random-sampling scheme covered all provinces and municipalities in China's mainland (data for Hong Kong, Macao and Taiwan are not available here). It has been demonstrated that with representative samples collected, the relative error decreased notably when the sample size was over 100. Compared with the last survey, the present work had significant improvements in interviewer training, field working experiences, questionnaire design, data collection and cleaning, ensuring the data quality and assurance (see Methods for details). However, some limitations still existed. First, there were potential biases and uncertainties with regard to some unreliable or partly missed information such as family income, household expenditure, the owning of modern appliances and vehicles, and months at home, due to privacy concerns. Second, the present study was a stratified random-sampling scheme but not a nationwide census. There were relatively higher uncertainties in the county- and/or village-level results, as the sample size was small. The national average and province-level results were representative and reliable. Analysis of the spatiotemporal results clearly demonstrated high diversity in the rural household energy mix and different transition paths, as well as the influence of factors such as resource availability, household characteristics like income, and policy.

Under rapid socio-economic development and urbanization, especially with the construction of a ‘Healthy China’, dramatic changes will occur in rural areas. These rapid changes will not be limited to increasing household incomes but will include other factors, such as an awareness of environmental protection and energy saving, better-educated people and more non-farming workers. Clean energy will thus be preferable in daily use if accessibility and affordability are not problems. Sustainable household energy should be an important part of national sustainable development. Unremitting efforts should be made to eliminate the use of dirty solid fuels and to promote the widespread availability and use of clean energy.

## METHODS

### Sampling and sample size

The data used in this study were collected during a national-scale survey of rural residents’ energy carriers and pollutant emissions. The survey was conducted in 2018 to collect basic data for 2017. The survey adopted stratified sampling methods at the provincial and municipal levels to ensure coverage of all provinces and municipalities, and random sampling below municipalities. This stratified random-sampling method with four levels attained good geographical representation. The sampling density was 0.43‰ for the nine populated provinces with high pollutant emission densities and 0.22‰ for the others. The survey covered all 31 provinces (excluding Hong Kong, Macao and Taiwan). In each municipal unit, several county units, based on the rural population, were extracted. In each selected county, unit villages and households were randomly selected. The sample size in each county was no more than 300. In each county, at least two villages were required, and in each village, a maximum household number of 80 was adopted. Therefore, the designed sample size was 50 000 households from 488 counties in 276 municipalities (a few adjacent municipalities were combined in the field survey due to very low sample sizes). Fuel weighing was applied to one village in a county unit, and the minimum number of households targeted was 2500. After the quality check, a final 56 556 valid questionnaires for the energy-mix survey, and 2615 fuel-weighing data points, were obtained.

### Energy-mix questionnaires and fuel-weighing

The questionnaire used in this study was updated from Tao *et al*. (2018) [17], and several available questionnaires used in international programs were customized to the habits and complexities of rural residential energy-carrier characteristics in China. Some questions and terms were revised in the questionnaire. The revised plan was used to carry out an on-site presurvey in rural Shandong, and the final questionnaire was formalized. The questionnaire survey included two aspects: section 1—personal and family-background information, and section 2—the amounts and fractions of different types of energy used. The personal and family-background information included the address, family size, income, demographics of family members, commodity energy expenditure, number of various energy-consuming tools and household appliances (lighting appliances and electrical appliances), number of different vehicles, and the number, location and types of stoves in each household. The energy information included the time-sharing fractions of various energy sources (coal, honeycomb briquettes, straw, corncobs, fuelwood, brushwood, animal manure, pipeline natural gas, LPG, biogas, electricity, solar, gasoline, diesel, etc.) for different activities (food cooking, subsidiary food preparation, water boiling, animal feed heating, space heating and cooling, etc.). Time-sharing data can effectively account for fuel stacking in residential energy use, which was one of the main limitations of the typical primary energy survey approach [17,27].

The energy structure of rural residential households was obtained by a face-to-face questionnaire survey conducted by trained investigators entering the households, and the questionnaires were completed by the investigators. The survey team underwent rigorous screening and training to ensure that the investigators fully understood the study objectives and the survey plan. The results of the questionnaire were checked by telephone and questionnaire screening. The survey process was monitored by recording the GPS information of the survey points and taking on-site photos to ensure the quality of the information. Written survey data were entered electronically by a third party using double-blind entry and machine-assisted verification. The entry accuracy rate was above 99%, and appropriate parameters were used for comprehensive verification and to eliminate records with unreasonable parameters.

The amounts of commercial energy used (coal, LPG and electricity) were asked for directly in the questionnaire. The daily consumption of solid fuels, including coal and biomass fuels, was obtained through a household fuel-weighing campaign. In the weighing campaign, the investigator weighed fuels on the first day and revisited the home after 24 hours to weigh the remaining fuels. Each household was surveyed for two consecutive days (weighed three times). The solid fuel consumption survey covered samples from both heating and non-heating periods, and the sample size in the heating and non-heating periods was 3:1. The results were adjusted for the family size. The amount of coal consumed and purchased every year was asked for in the questionnaire survey. The self-reported results varied largely across different households. The provincial coal consumption calculated from the self-reported data was positively correlated with that from the fuel-weighing results (*r* = *0.868*, *p* < 0.001), but the self-reported results were much lower than the fuel-weighing results.

### Data analysis

Data analysis and statistical tests were conducted using Excel and SPSS. A significance level of 0.05 was adopted. The results at the household level were weighted by the household numbers to obtain provincial and national averages. The uncertainties associated with the provincial and national average results were addressed by running Monte Carlo simulations 10 000 times. Provincial socio-economic information was obtained from the national statistical yearbook, and the HDD data were calculated from the recorded ambient temperature using the method described by Chen *et al*. (2016) [36]. The factors influencing the fraction of clean energy sources (gas and electricity) in cooking and heating activities, and the total energy consumption amount of fuels and electricity, were studied by using the double-hurdle model. The independent variables tested included location, family size, number of energy sources, number of stoves, household income, HDDs, male residents at home, highest education level, gender of the cook, education level of the main cook, occupation of the main cook, household space area and age of the house.

## DATA AVAILABILITY

Data associated with the study are available upon request.

## Supplementary Material

nwac050_Supplemental_File
