# Supplementary material for: Substantial transition to clean household energy mix in rural China
Source: Natl Sci Rev. 2022 Mar 14;9(7):nwac050. doi: 10.1093/nsr/nwac050 (PMC9283105; doi:10.1093/nsr/nwac050)

**Supporting information**

**Substantial transition to clean household energy mix in rural households in China**

Guofeng Shen**^1^**, Rui Xiong**^1^**, Yanlin Tian**^1,2^**, Zhihan Luo**^1^**, Bahabaike Jiangtulu**^3^**, Wenjun Meng**^1^**, Wei Du**^4^**, Jing Meng**^5^**, Yuanchen Chen**^6^**, Bing Xue**^7^**, Bin Wang**^3, 8^**, Yonghong Duan**^9,^**^ꝭ^, Jia Duo**^10, 11, 12,^**^ꝭ^, Fenggui Fan**^13,^**^ꝭ^, Lei Huang**^14,^**^ꝭ^, Tianzhen Ju**^15,^**^ꝭ^, Fenggui Liu**^16, 17,^**^ꝭ^, Shunxin Li**^18,^**^ꝭ^, Xianli Liu**^19,^**^ꝭ^, Yungui Li**^20,^**^ꝭ^, Wangmo**^22,^**^ꝭ^, Ying Nan**^22,^**^ꝭ^, Bo Pan**^23,^**^ꝭ^, Yanfang Pan**^24,^**^ꝭ^, Lizhi Wang**^25,^**^ꝭ^, Eddy Zeng**^26,^**^ꝭ^, Chao Zhan**^27,^**^ꝭ^, Yilin Chen**^28^**, Huizhong Shen**^28^**, Hefa Cheng**^1^**, Shu Tao**^1, 28,^**^*^

1. *College of urban and Environmental Sciences, Peking University, Beijing 100871, China*
2. *Collaborative Innovation Center of Atmospheric Environment and Equipment Technology, Jiangsu Key Laboratory of Atmospheric Environment Monitoring and Pollution Control (AEMPC), Nanjing University of Information Science & Technology, Nanjing 210044, China*
3. *Institute of Reproductive and Child Health, Peking University, Beijing 100191, China.*
4. *Laboratory of Geographic Information Science, School of Geographic Sciences, East China Normal University, Shanghai 200241, China*
5. *The Bartlett School of Sustainable Construction, University College London, London WC1E 7HB, UK*
6. *College of Environment, Zhejiang University of Technology, Hangzhou 310014, China*
7. *Institute of Applied Ecology, Chinese Academy of Sciences, Shenyang 110016, China*
8. *Department of Epidemiology and Biostatistics, School of Public Health, Peking University, Beijing 100191, China*
9. *College of Resources and Environment, Shanxi Agricultural University, Jinzhong 030801, China*
10. *Xinjiang Key Laboratory of Environmental Pollution and Bioremediation, Xinjiang Institute of Ecology and Geography, Chinese Academy of Science, Urumqi 830011, China.*
11. *National Engineering Technology Research Center for Desert-Oasis Ecological Construction, Xinjiang Institute of Ecology and Geography, Chinese Academy of Sciences, Urumqi 830011, China.*
12. *University of Chinese Academy of Sciences, Beijing 100049, China.*
13. *School of Geography and Tourism, Anhui University, Wuhu 241000, China*
14. *School of Environment, Nanjing University, Nanjing 210033, China*
15. *College of Geography and Environmental Science, Northwest Normal University, Lanzhou 730070, China*
16. *College of Geographical Science, Qinghai Normal University, Xining 81008, China*
17. *Academy of Plateau Science and Sustainability, Xining 81008, China*
18. *College of Chemistry, Chemical Engineering and Environment, Minnan Normal University, Zhangzhou 363000, China*
19. *School of Environmental Science and Engineering, Hubei Polytechnic University, Huangshi 435003, China*
20. *Department of Environmental Engineering, Southwest University of Science and Technology, Mianyang 621010, China*
21. *College of Food Science, Tibet Agricultural and Animal Husbandry University, Linzhi 860000, China*
22. *College of Geography and Ocean Sciences, Yanbian University, Yanji 133002, China*
23. *Faculty of Environmental Science& Engineering, Kunming University of Science and Technology, Kunming 650500, China.*
24. *College of Geography and Environmental Science, Henan University, Kaifeng 475001, China*
25. *College of Ecology and Environment, Hainan University, Haikou 570228, China*
26. *School of Environment, Jinan University, Guangzhou 510632, China*
27. *Institute of Coastal Research, Ludong University, Yantai 264025, China*
28. *College of Environmental Science and Technology, Southern University of Science and Technology, Shenzhen 518055, China*

ꝭ. Equal contribution and in the alphabetical order of the first name

*. Corresponding to [taos@pku.edu.cn](mailto:taos@pku.edu.cn)

**Figure s1**. Frequency distribution of the energy type number per household (A), and the relationship between the number of energy type per household and the number of stoves per household (B). Data for Hong Kong, Macao, and Taiwan province are not available in this study.


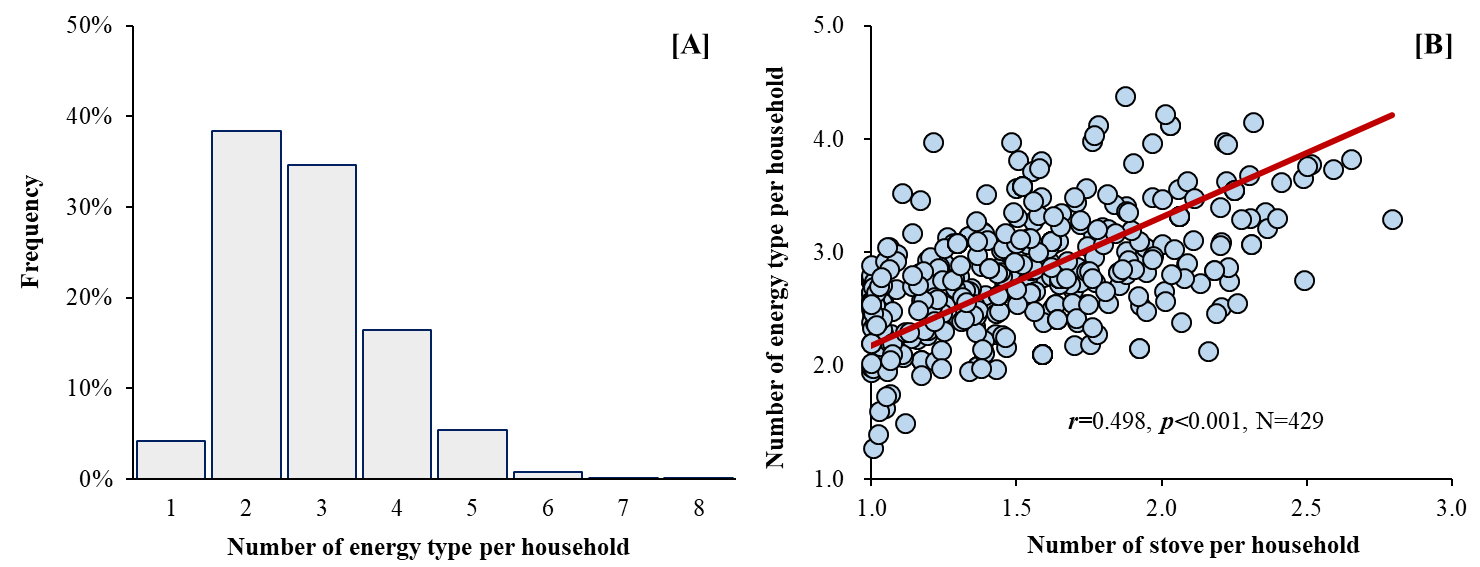


**Figure s2**. Percentage of households primarily using different energy sources for cooking (A) and heating (B) in rural China in 2017. Data for Hong Kong, Macao, and Taiwan province are not available in this study.

**Figure s3**. Plots of province-level time-sharing fraction of clean energy sources for cooking (***F****_C_*) and that for heating (***F****_H_*) in 2017. Data for Hong Kong, Macao, and Taiwan province are not available in this study.

**Figure s4**. Mass fractions of coal, biomass, and gas fuels in activities of cooking, heating, and animal foods. Data for Hong Kong, Macao, and Taiwan province are not available in this study.


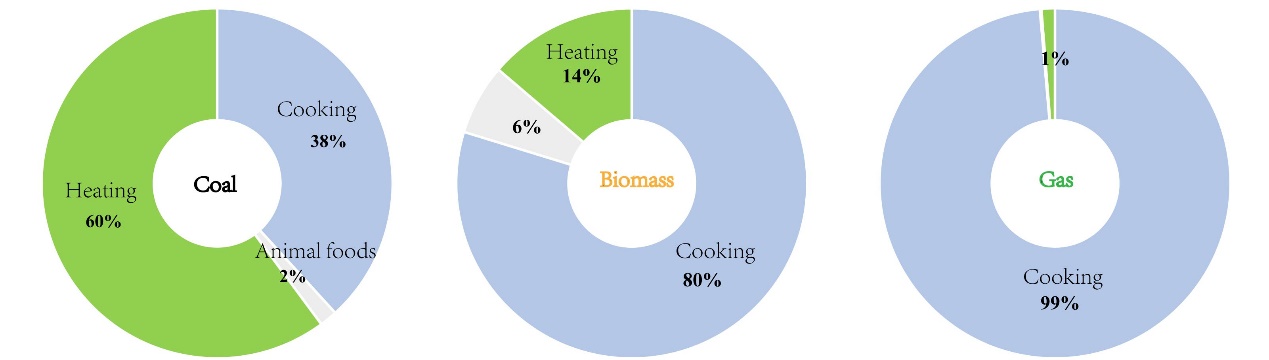


**Figure s5**. Coal, biomass, and gas consumption amounts, in KJ, for each province in China. Data for Hong Kong, Macao, and Taiwan province are not available in this study.


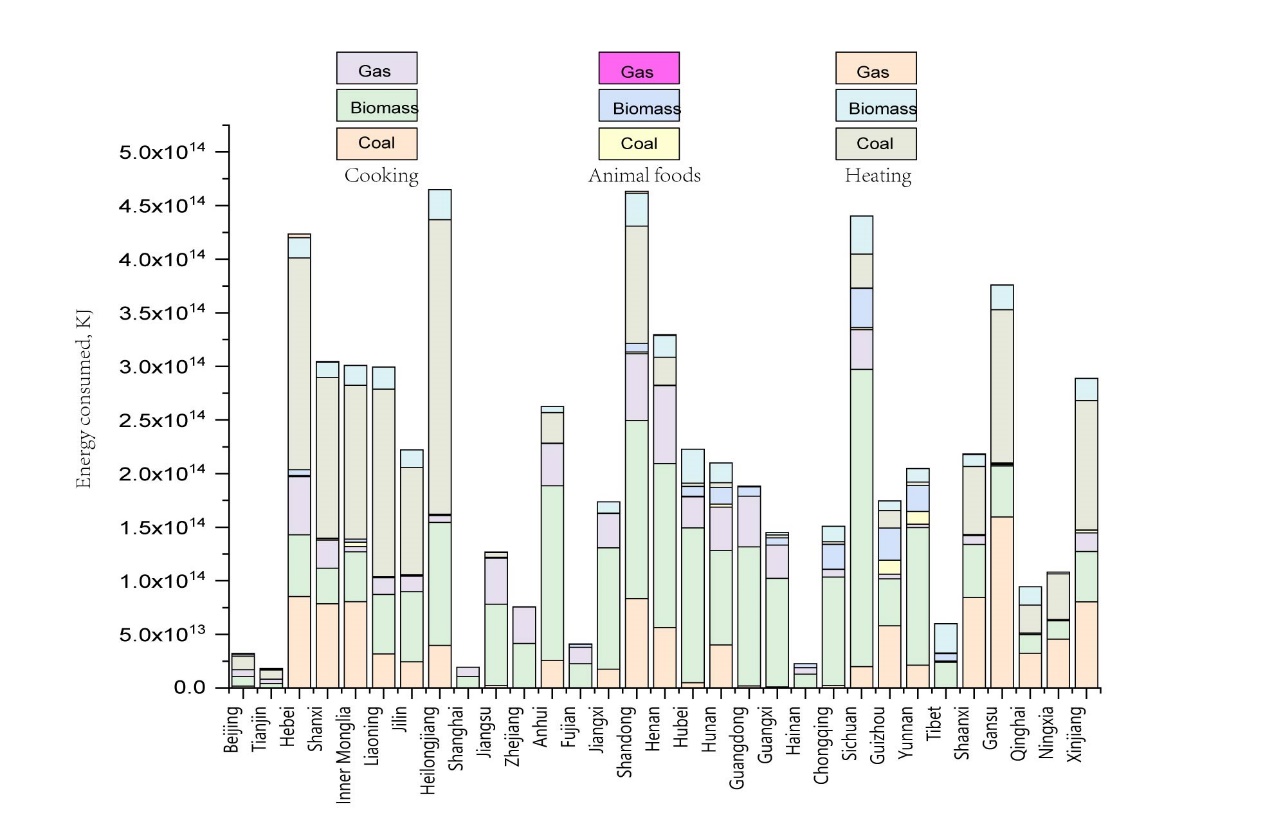


**Figure s6**. (A) relationship between fuel consumption and the average number of households in provinces, and (B) dependence of the per household fuel consumption on the percentage of heating energy, and on the percentage of solid fuels consumed.


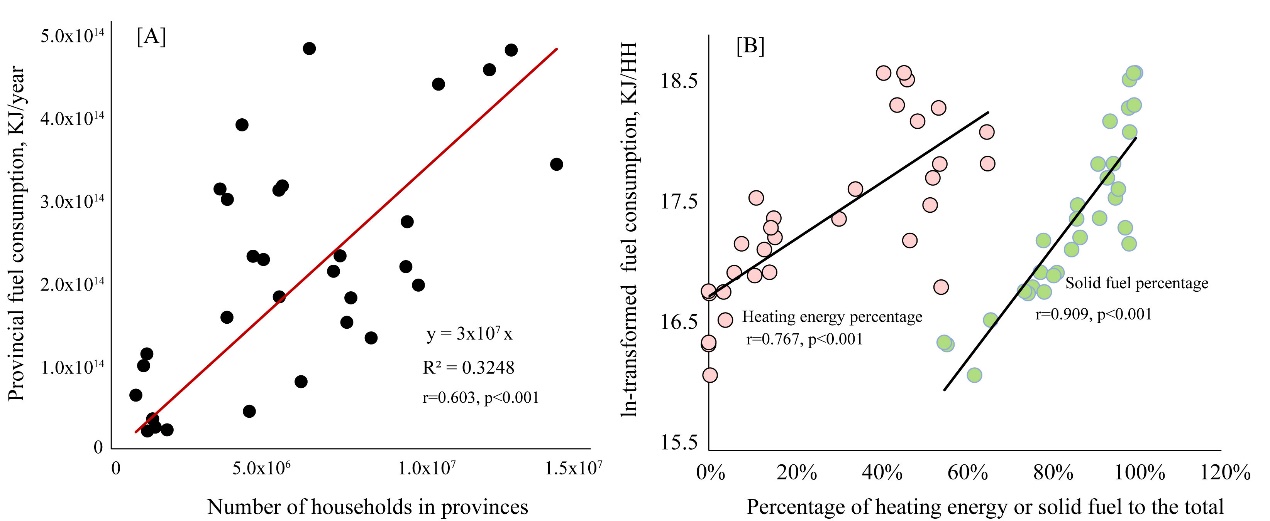

Supplement: nwac050_Supplemental_File [file nwac050_supplemental_file.docx]
